# BDNF: A Key Factor with Multipotent Impact on Brain Signaling and Synaptic Plasticity

**DOI:** 10.1007/s10571-017-0510-4

**Published:** 2017-06-16

**Authors:** Przemysław Kowiański, Grażyna Lietzau, Ewelina Czuba, Monika Waśkow, Aleksandra Steliga, Janusz Moryś

**Affiliations:** 1 Department of Anatomy and Neurobiology, Medical University of Gdansk, 1 Debinki Street, 80-211 Gdańsk, Poland; 2 Department of Health Sciences, Pomeranian University of Slupsk, 64 Bohaterów Westerplatte Str., 76-200 Słupsk, Poland

**Keywords:** BDNF, Cognition, Development, Neurotrophin, Synaptic plasticity

## Abstract

Brain-derived neurotrophic factor (BDNF) is one of the most widely distributed and extensively studied neurotrophins in the mammalian brain. Among its prominent functions, one can mention control of neuronal and glial development, neuroprotection, and modulation of both short- and long-lasting synaptic interactions, which are critical for cognition and memory. A wide spectrum of processes are controlled by BDNF, and the sometimes contradictory effects of its action can be explained based on its specific pattern of synthesis, comprising several intermediate biologically active isoforms that bind to different types of receptor, triggering several signaling pathways. The functions of BDNF must be discussed in close relation to the stage of brain development, the different cellular components of nervous tissue, as well as the molecular mechanisms of signal transduction activated under physiological and pathological conditions. In this review, we briefly summarize the current state of knowledge regarding the impact of BDNF on regulation of neurophysiological processes. The importance of BDNF for future studies aimed at disclosing mechanisms of activation of signaling pathways, neuro- and gliogenesis, as well as synaptic plasticity is highlighted.

## Introduction

In 1989, Yves-Alain Barde and Hans Thoenen isolated brain-derived neurotrophic factor (BDNF) from pig brain, and shortly afterwards its biochemical structure was revealed (Barde et al. 1982; Leibrock et al. 1989). The human BDNF gene consists of 11 exons, and its different splicing enables formation of transcripts specific to various tissues and responsive to different stimuli (Pruunsild et al. 2007). The conservative structure of BDNF, with 85.9–100 % identity among genes of various vertebrates and humans, determines its physiological function, to a large extent independently of the stage of phylogenetic development (Yeh et al. 2015). BDNF is a member of the neurotrophin family, which also includes nerve growth factor (NGF), neurotrophin 3 (NT3), and neurotrophin 4 (NT4) (Hohn et al. 1990; Ip et al. 1992; Maisonpierre et al. 1990; Rosenthal et al. 1990).

A constantly growing body of evidence indicates involvement of BDNF in a wide range of neurophysiological processes. This can be explained based on its characteristic pattern of synthesis, which involves several biologically active isoforms that interact with different receptors, thereby controlling numerous signaling pathways. BDNF is present in nearly all brain regions (Hofer et al. 1990; Yan et al. 1997). Its function differs depending on both the stage of brain development as well as the neuronal, glial, or vascular constituents of the brain tissue. The most important functions of BDNF include developmental processes, regulation of neuro-, glio-, and synaptogenesis, neuroprotection, and control of short- and long-lasting synaptic interactions that influence mechanisms of memory and cognition (for review, see Foltran and Diaz 2016; Gonzalez et al. 2016; Park and Poo 2013; Sasi et al. 2017).

In this review, we present current views on BDNF synthesis and elaborate its active isoforms, their interactions with specific receptors, and hypotheses explaining the role of BDNF in regulation of signaling pathways involved in developmental processes, neuroprotection, and synaptic plasticity.

## Functionally Active BDNF Precursor Isoforms are Produced in the Course of Multistage Synthesis

**Fig. 1:**
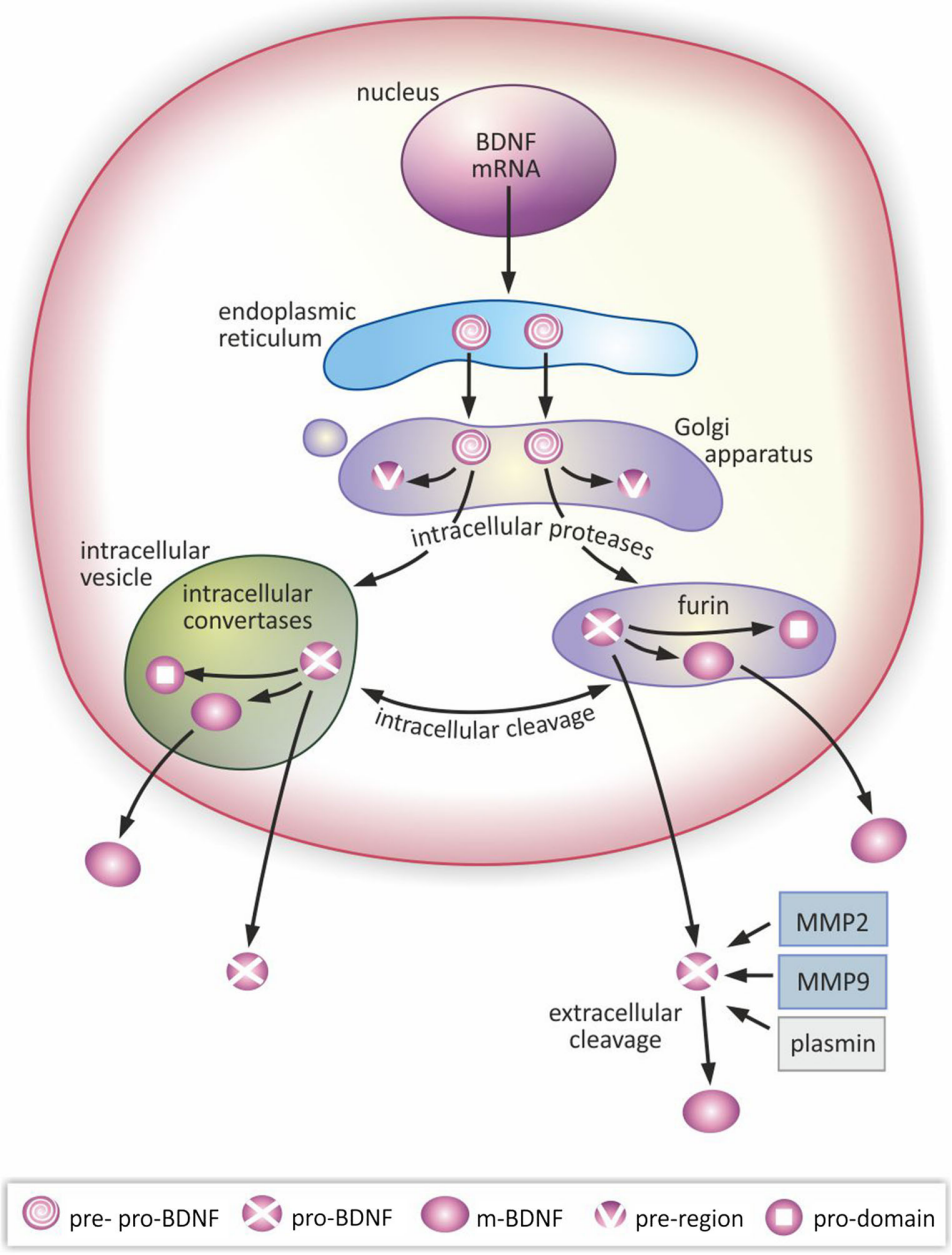
Schematic presentation of synthesis and maturation of BDNF. BDNF synthesis and maturation is a multistage sequence of intra- and extracellular processes. In the intracellular pathway, the pre-pro-BDNF precursor sequence is produced in the endoplasmic reticulum and transported to the Golgi apparatus. In the course of intracellular cleavage, the pre-region sequence is removed, resulting in formation of immature proneurotrophin isoform of BDNF (pro-BDNF). Further, after removal of the pro-domain sequence, the mature isoform of BDNF (m-BDNF) is produced. Intracellular cleavage leading to formation of m-BDNF also occurs in intracellular vesicles, allowing transport of this neurotrophin to axonal terminals and subsequent release into the extracellular space, via presynaptic membrane. Processing of BDNF is accomplished by intracellular proteases, regulated convertases, and furin. As a result, both pro-BDNF and m-BDNF isoforms are released into the extracellular space. In the extracellular pathway, pro-BDNF released into the extracellular space is processed by metalloproteinases 2 and 9 (MMP2 and MMP9), plasmin, and extracellular proteases. Consequently, functionally effective isoforms of m-BDNF and pro-BDNF can be found in the extracellular space. *BDNF* brain-derived neurotrophic factor, *m-BDNF* mature isoform of BDNF, *MMP2* metalloprotease 2, *MMP9* metalloprotease 9, *pre-pro-BDNF* primary, uncleaved precursor form of BDNF, *pre-region* region of precursor sequence, *pro-BDNF* proneurotrophin isoform of BDNF after cleavage of pre-region precursor sequence, *pro-domain* sequence cleaved from proneurotrophin isoform of BDNF when it becomes mature BDNF

Synthesis and maturation of BDNF is a multistage process, involving formation of several precursor isoforms. The BDNF protein is synthesized and folded in the endoplasmic reticulum as a precursor form, pre-pro-BDNF (Fig. 1) (Foltran and Diaz 2016; Lu 2003). Upon translocation to the Golgi apparatus, the signal sequence of the pre-region is cleaved, resulting in formation of the precursor proneurotrophin isoform of BDNF (pro-BDNF). This protein consists of 129 amino acids containing an N-terminal pro-domain and 118 amino acids with a C-terminal mature domain (Mowla et al. 2001; Rosenthal et al. 1991). The pro-BDNF is further cleaved to reach the mature isoform (m-BDNF) (Foltran and Diaz 2016; Mizui et al. 2016). Intracellular proteolytic cleavage of pro-BDNF may occur in the trans-Golgi network by constitutively released furin, or in intracellular secretory vesicles by regulated convertases (Lu et al. 2005). Extracellular cleavage of pro-BDNF is dependent on plasmin (Pang et al. 2004) and matrix metalloproteases 2 and 9 (MMP2 and MMP9) (Hwang et al. 2005; Mizoguchi et al. 2011; Vafadari et al. 2016). Secretion of m-BDNF and pro-BDNF into the extracellular space enables their physiological action. The characteristic pattern of neurotrophin synthesis provides, on the one hand, an opportunity to control the enzymatic activity involved in generation of BDNF isoforms, while on the other, it can explain their role in regulation of several physiological processes, frequently having opposite final effects.

Depending on the cell type, BDNF secretion can be constitutive or activity dependent (Mowla et al. 2001). In neuronal cells, both pro-BDNF and m-BDNF are released following cellular membrane depolarization (Conner et al. 1997; Dieni et al. 2012; Yang et al. 2009). The above-mentioned mechanisms maintain a dynamic balance between various isoforms of BDNF. The ratio of pro-BDNF to m-BDNF varies between particular stages of brain development and regions. While in the early postnatal period higher concentration of pro-BDNF is reported, m-BDNF prevails in adulthood (Yang et al. 2014). Consequently, pro-BDNF may be regarded as an important factor modulating brain function, especially in its development, whereas m-BDNF reveals its significance for processes occurring in adulthood, such as neuroprotection and synaptic plasticity.

Apart from the two above-mentioned isoforms, the functioning of BDNF is significantly affected by the single-nucleotide polymorphism of methionine (Met) to valine (Val) substitution at position 66 within the BDNF gene in the pro-domain encoding region (Egan et al. 2003). According to some recently published data, the Met66 pro-domain variant can be regarded as another ligand with independent significance for BDNF communication (for review, see Hempstead 2015).

In conclusion, the multistage pattern of BDNF synthesis and maturation facilitates the contribution of its isoforms to regulation of processes that occur at different stages of brain development, in various cellular populations, as well as in several functional systems.

## The BDNF Isoforms Interact with Different Types of Receptors, Triggering a Wide Range of Signaling Cascades

**Fig. 2:**
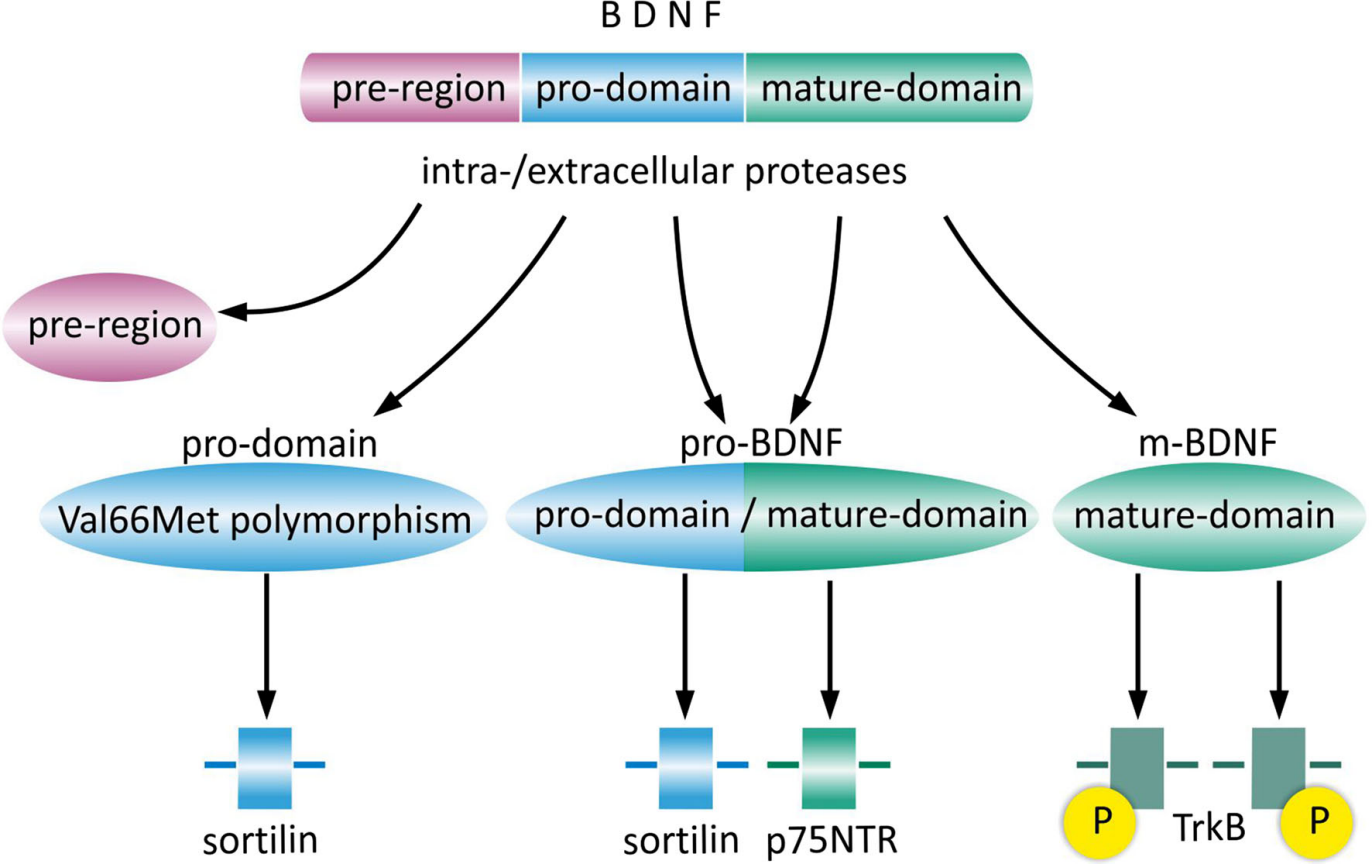
Interaction of BDNF isoforms with specific receptors. As a consequence of intra- or extracellular cleavage, the primary sequence of pre-pro-BDNF is divided into functionally active isoforms of pro-domain, pro-BDNF, and m-BDNF, each of which exhibits characteristic affinity to a specific type of receptor. The BDNF pro-domain binds preferentially to the sortilin receptor. Although the Val66Met polymorphism of the pro-domain does not exclude its binding with sortilin, the receptor affinity and functional effects resulting from Val66 or Met66 pro-domain binding are characteristic for each of them. The pro-BDNF isoform consisting of two sequences (pro-domain and mature domain) interacts with specific receptors (sortilin and p75NTR, respectively). The mature domain of BDNF, being the only constituent of the m-BDNF isoform, exhibits highest affinity for the TrkB receptor, which when stimulated undergoes homodimerization and autophosphorylation. *P* phosphate group, *p75NTR* p75 neurotrophin receptor, *sortilin* sortilin-related vacuolar protein sorting 10 protein (Vps10p)-domain sorting receptor 2, *TrkB* tyrosine kinase B receptor, *Val66Met polymorphism* polymorphism of BDNF pro-domain resulting from methionine to valine substitution at position 66 within the BDNF gene in the pro-domain encoding region

Pro-BDNF interacts preferentially with the p75 neurotrophin receptor (p75NTR), a member of the tumor necrosis factor (TNF) receptor family, through its mature domain, and with the sortilin receptor or other vacuolar protein sorting 10 protein (Vps10p) of the sorting receptor family, through its pro-domain (Fig. 2) (for review, see Anastasia et al. 2013; Teng et al. 2005). m-BDNF binds the tyrosine kinase B (TrkB) receptor, belonging to the tropomyosin-related kinase (Trk) family of receptor tyrosine kinases (Chao and Hempstead 1995; Ebendal 1992; Reichardt 2006). In resting form, both types of receptor are located in the membrane of intracellular vesicles. Stimulation with Ca^2+^, cyclic adenosine monophosphate (cAMP), or electrical impulse initiates their transfer and fusion with the cellular membrane (Du et al. 2000; Meyer-Franke et al. 1998).

Activation of p75NTR requires formation of complexes within the cellular membrane which consist of different types of precursor neurotrophins and signaling adaptors. This allows signal transfer and activation of transduction pathways (Chao and Hempstead 1995). The changing composition of such membrane complexes is responsible for the wide spectrum of functions controlled, often with opposing character of final effect, which can vary from supporting neuronal survival to inhibition of growth, or even apoptotic death (Teng et al. 2005).

**Fig. 3:**
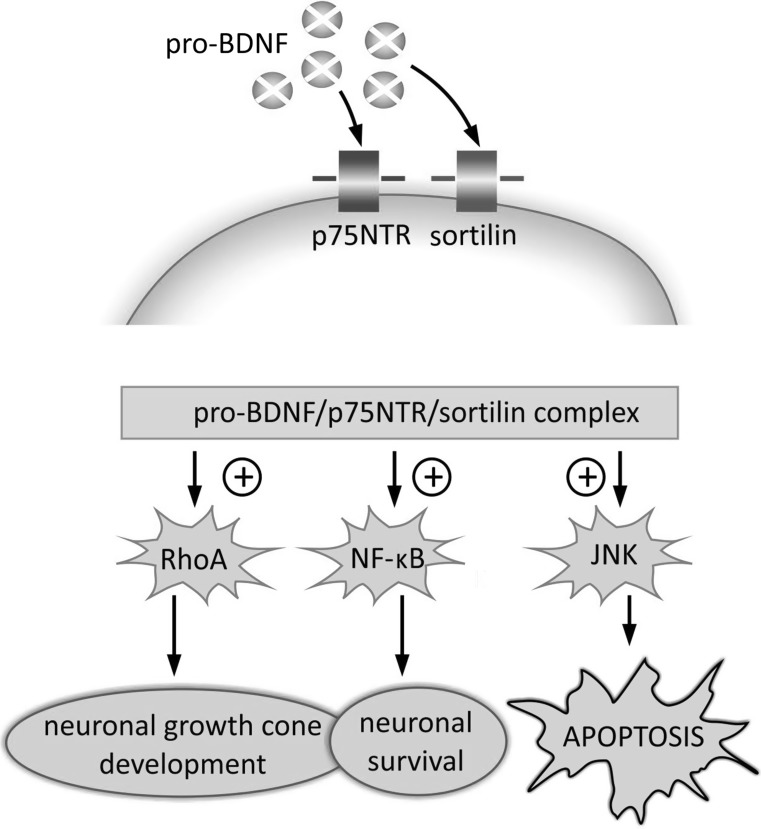
Intracellular signaling cascades activated by interaction of pro-BDNF isoform with p75NTR and sortilin receptors. The sequences of pro-domain and mature domain (m-BDNF), which form the proneurotrophin isoform (pro-BDNF), reveal preferential affinity for sortilin and p75NTR, respectively. This results in formation of pro-BDNF/p75NTR/sortilin binding complex and triggering of signaling pathways related with RhoA, NF-κB and JNK, which promotes processes leading to neuronal development and survival, but also to programmed cell death. *JNK* c-Jun amino terminal kinase, *NF-κB* nuclear factor kappa B, *p75NTR* p75 neurotrophin receptor, *pro-BDNF* proneurotrophin isoform of BDNF, *RhoA* Ras homolog gene family member A, *sortilin* sortilin-related Vps10p-domain sorting receptor 2

Activation of receptors and formation of specific complexes within the cellular membrane triggers several signaling pathways. The pro-BDNF/p75NTR/sortilin binding complex initiates signaling cascades leading to activation of c-Jun amino terminal kinase (JNK), Ras homolog gene family member A (RhoA), and nuclear factor kappa B (NF-κB) (Fig. 3) (Anastasia et al. 2013; Reichardt 2006). The functional implications resulting from activation of the above-mentioned signaling cascades have been systematically studied.

The JNK-related pathway, which is activated by the pro-BDNF/p75NTR/sortilin complex, triggers neuronal apoptosis (Anastasia et al. 2013; Teng et al. 2005). This mechanism of cell elimination has been confirmed by reports indicating high level of p75NTR expression during brain development and posttraumatic recovery (Barker 1998; Martínez-Murillo et al. 1993; Roux et al. 1999). Activation of the RhoA-dependent signaling pathway is reported to regulate neuronal growth cone development and motility (Reichardt 2006). Finally, p75NTR-dependent activation of NF-κB supports processes promoting neuronal survival and maintenance of their adequate number during brain development (Reichardt 2006).

Hence, pro-BDNF binding to specific receptors triggers signaling pathways which can determine neuronal fate via promoting their death or survival. It can also determine the pathway of further development and morphological differentiation. Neurons influenced by high level of pro-BDNF or remaining under low concentration of m-BDNF prevalently undergo elimination (Bamji et al. 1998). This pattern of pro-BDNF-related regulation can occur during both brain development and postlesion recovery.

**Fig. 4:**
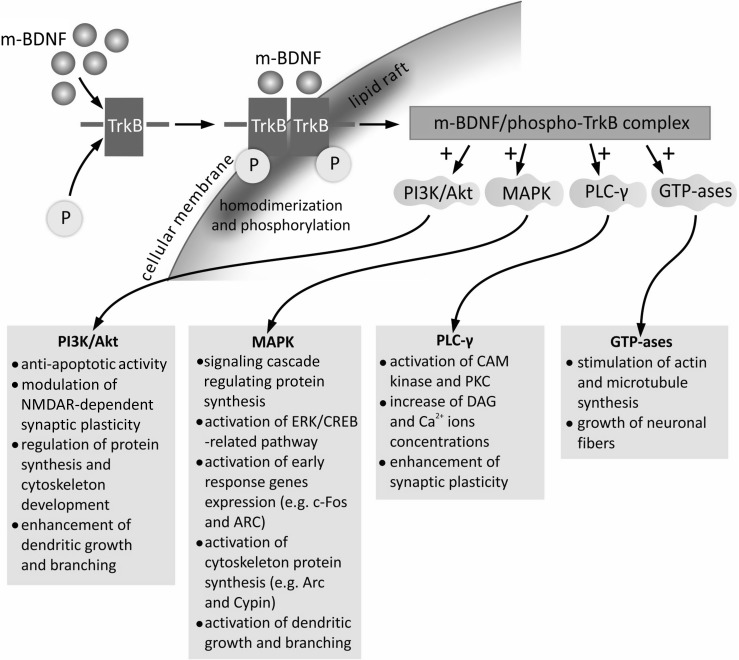
Intracellular signaling cascades activated by interaction of m-BDNF isoform with TrkB receptor. Binding of the m-BDNF isoform to TrkB receptor triggers its homodimerization and phosphorylation with subsequent translocation to cellular membrane lipid rafts, rich in cholesterol and sphingolipids. The m-BDNF/TrkB receptor complex triggers signaling pathways associated with activation of PI3K, MAPK, PLC- γ, and GTP-ases of the Rho family. *CAM kinase* Ca^2+^-calmodulin-dependent protein kinase, *CREB* cAMP response element-binding protein, *DAG* 1,2-diacylglycerol, *ERK* extracellular-signal-regulated kinase, *GTP-ases* guanosine triphosphate hydrolases, *MAPK* mitogen-activated protein kinase, *m-BDNF* mature isoform of BDNF, *NMDAR N*-methyl-d-aspartate receptor, *P* phosphate group, *PI3K* phosphatidylinositol 3-kinase, *PKC* protein kinase C, *PLC-γ* phospholipase C-γ, *Rho* Ras homolog gene family member, *TrkB* tyrosine kinase B receptor

The m-BDNF isoform, binding with the high-affinity TrkB receptor, initiates its dimerization and autophosphorylation of intracellular tyrosine residues, which results in formation of phosphorylated-TrkB receptor (Fig. 4) (Kaplan and Miller 2000). An important process determining the stimulatory effect of the m-BDNF/TrkB receptor complex is its translocation toward cellular membrane lipid rafts, i.e., microdomains rich in cholesterol and sphingolipids (Suzuki et al. 2004). Phosphorylated-TrkB activates several enzymes: phosphatidylinositol 3-kinase (PI3K), mitogen-activated protein kinase (MAPK), phospholipase C-γ (PLC-γ), and guanosine triphosphate hydrolases (GTP-ases) of the Ras homolog (Rho) gene family (Gonzalez et al. 2016; Huang and Reichardt 2003; Minichiello 2009). All of these trigger signaling cascades with determined cellular functions.

The PI3K/Akt-related pathway exerts antiapoptotic and prosurvival activity and modulates *N*-methyl-d-aspartate receptor (NMDAR)-dependent synaptic plasticity (Baydyuk and Xu 2014; Gonzalez et al. 2016; Park and Poo 2013). The PI3K/Akt/mTOR cascade, through regulation of protein synthesis and cytoskeleton development, enhances dendritic growth and branching (Jaworski et al. 2005; Kumar et al. 2005).

The MAPK/Ras signaling cascade regulates protein synthesis during neuronal differentiation (Reichardt 2006). MAPK-related signaling is also required for activation of extracellular-signal-regulated kinase 1/2 (ERK 1/2) and cAMP response element-binding protein (CREB) (Finkbeiner et al. 1997; Xing et al. 1998). This pathway is critical not only for early response gene expression (e.g., c-Fos and ARC), but also for cytoskeleton protein synthesis (e.g., Arc and cypin) (Gonzalez et al. 2016), as well as dendritic growth and branching in hippocampal neurons (Kwon et al. 2011; Segal 2003).

The PLC-γ-dependent pathway evokes activation of Ca^2+^-calmodulin-dependent protein kinase (CAM kinase) and protein kinase C (PKC), which subsequently increase the 1,2-diacylglycerol (DAG) and Ca^2+^ ion concentrations (Alcántara et al. 1997; Minichiello 2009). The PKC-dependent pathway is reported to enhance synaptic plasticity (Reichardt 2006). BDNF/TrkB complex-initiated activation of GTP-ases, representing the Rho family, stimulates actin and microtubule synthesis, which results in growth of neuronal fibers (Gonzalez et al. 2016).

In summary, the specific role of BDNF in regulation of numerous physiological processes in the brain is a consequence of interaction of its isoforms with different types of receptor. This allows triggering of signaling pathways that are critical for maintaining a dynamic balance between stimulating and inhibitory effects exerted upon processes of brain development, synaptic plasticity, and brain regeneration after damage. Understanding the physiological function of BDNF may be critical for further research on regulatory mechanisms of cellular signaling. Disorders of BDNF synthesis, resulting in dysfunction of regulated signaling cascades, may be responsible for triggering several pathological processes.

## The BDNF Isoforms Positively and Negatively Contribute to Maintenance of Brain Homeostasis

One of the most important features of BDNF synthesis is the elaboration of several functionally active isoforms, such as pro-BDNF, m-BDNF, pro-domain sequence, and Val66Met polymorphic pro-domain variant. All of these exert characteristic influences on numerous physiological processes. The positive or negative impact exerted by these isoforms enables precise control of the dynamic balance which is essential for maintenance of physiological homeostasis. Additional factors, such as brain developmental stage, brain structure, targeted cellular population, and environmental factors, are also important for this type of regulation.

During brain development, the most important issues related to BDNF isoforms include neuro-, glio-, and synaptogenesis, regulation of cell death, and elimination of improperly formed connections. In adulthood, the prevailing processes enhance the efficiency of stimulus transmission and synaptic plasticity, which support memory and cognition.

The biological function of pro-BDNF has been a subject of discussion for many years (for review, see Gonzalez et al. 2016; Hempstead 2015; Mizui et al. 2016). Initially, it was regarded rather as an inactive protein. However, recently it has been characterized as an independent ligand, demonstrating specific biological activity (Anastasia et al. 2013; Dieni et al. 2012). Among the most important functions of pro-BDNF, one can mention promotion of apoptosis and its negative influence on neuronal remodeling, reflected by growth cone retraction and dendritic spine shrinkage (Gehler et al. 2004; Yamashita et al. 1999). Due to the reduction in the number of neurons and deterioration of synaptic function, these processes contribute to long-term depression (LTD), as revealed in hippocampal neurons (Park and Poo 2013; Woo et al. 2005; Yang et al. 2014). The physiological significance of all these apparently negative processes can be explained by the reduction of an excessive number of maturing neurons, elimination of damaged or malfunctioning cells, as well as elimination of abnormal connections that are ineffective for formation of synaptic plasticity, memory, and cognition.

In contrast to pro-BDNF, m-BDNF supports developmental processes of neuro- and gliogenesis (Gonzalez et al. 2016; Vilar and Mira 2016), neurite outgrowth, dendritic arborization, and dendritic spine formation (Encinas et al. 1999; Yamada et al. 2001). The physiological effect of m-BDNF is mainly related to maintenance of synaptic strength and decreased excitability of hippocampal GABA-ergic interneurons, together enhancing hippocampal long-term potentiation (LTP) (Leal et al. 2015; Park and Poo 2013). Hence, the physiological function of m-BDNF is linked to enhancement of developmental processes, as well as to processes in adulthood that require increased brain activity and support efficient stimulus transmission in the synaptic system, finally resulting in improved memory and cognition.

The results of early studies did not reveal the physiological activity of BDNF pro-domain, but more recent data have shed more light on its potential function (Anastasia et al. 2013). The concentration of BDNF pro-domain rises during adolescence and adulthood, following the increase of m-BDNF, which may provide evidence of its functional significance. It is released from neurons after depolarization, as an activity-dependent ligand with definite physiological properties (Dieni et al. 2012). BDNF pro-domain interacts with the sortilin receptor. However, it reveals different bioactivity depending on the pro-domain variant (Val66 or Met66), most probably due to interaction via different residues. The molecular mechanism of Val66Met polymorphism in BDNF pro-domain relies on its impaired interaction with the sortilin receptor (sortilin-related Vps10p-domain sorting receptor 2) (Chen et al. 2005). This has been postulated to be responsible for altered spatial conformation, changed intracellular sorting and trafficking, as well as impaired release of neurotrophin into the synaptic cleft (Anastasia et al. 2013; Chen et al. 2004, 2005). The consequences of these effects can include changes in neuronal growth cone morphology, or their retraction, as well as impaired synaptic plasticity. There is well-documented evidence indicating that these processes are responsible for initiating characteristic phenotypical manifestations that are critical in the development of several neurodegenerative disorders and processes related to episodic cognition and memory disturbances, increased risk of depression, and anxiety development (Dincheva et al. 2012; Egan et al. 2003; Isackson et al. 1991; Soliman et al. 2010; Verhagen et al. 2010).

## The Neuroprotective Effects of BDNF are Related to Modulation of NMDAR/Ca^2+^-Dependent Signaling

m-BDNF-dependent neuroprotection regulates the dynamic balance between prosurvival NMDAR-dependent synaptic signaling and death-inducing extrasynaptic communication (Lau et al. 2015). The m-BDNF/TrkB receptor complex triggers the synaptic NMDAR/Ca^2+^-driven signaling cascade, leading to increased expression of inhibin β-A (activin A in homodimer form) (Lau et al. 2015; Zhang et al. 2009). The postulated neuroprotective effect is related to elimination of glutamatergic excitotoxicity, resulting from inhibition of the extrasynaptic NMDAR-mediated Ca^2+^ influx. This prevents the mitochondrial dysfunction and apoptotic cell death observed in the course of neurodegenerative diseases (Zuccato and Cattaneo 2009).

The neuroprotective effect can also be achieved due to synaptic NMDAR stimulation and subsequent increase of the nuclear Ca^2+^ influx, which results in activation of CREB and increased expression of genes coding proteins involved in neuroprotection (Bading 2013; Zhao et al. 2017). The above-mentioned mechanisms enable determination of the neuronal fate during brain development and in adulthood. Impairment of these mechanisms has been demonstrated in neurodegenerative diseases, such as Huntington’s and Alzheimer’s (Zuccato and Cattaneo 2009).

## BDNF Contributes to Neurogenesis by Modulating Its Advanced Stages

Among the most important aspects of neurogenesis, one could mention maintenance of an adequate number of proliferating neural progenitors and conditions that enable their further growth and differentiation (Dwyer et al. 2016; Germain et al. 2010). Brain development, on the one hand, relies on coordinated processes of neuro- and gliogenesis, formation of neuronal projections, and synaptogenesis, but on the other hand, is related to programmed cell death and elimination of improperly formed connections, together resulting in the formation of the functionally and morphologically adjusted structure of the adult brain. In spite of intensive studies, the role of BDNF in these processes remains unclear, and available data frequently remain contradictory.

Whereas the prosurvival function of BDNF exerted upon neurons in the developing brain is not evident, it has been reported to enhance survival of neurons in the adult brain after trauma or during neurodegeneration (Ebadi et al. 1997). In vivo studies showed that BDNF is involved in regulation of neurogenesis in the adult hippocampus (Katoh-Semba et al. 2002; Lee et al. 2002; Scharfman et al. 2005). However, some authors question the role of this neurotrophin in survival of new neurons in the adult dentate gyrus (Vilar and Mira 2016).

In the light of current views, based on the results of in vivo studies, the role of BDNF in neurogenesis could be summarized more as a differentiating factor, rather than a survival factor. An accumulating body of evidence indicates action of BDNF during the later stages of neurogenesis (Bergami et al. 2008; Chan et al. 2008; Wang et al. 2015). Binding of BDNF to TrkB receptor stimulates neuronal differentiation and dendritic morphogenesis in the subgranular zone of the hippocampus, confirming its function during advanced stages of neurogenesis. While BDNF deficit does not result in a significant decrease in the number of neurons, it does cause inhibition of dendritic arborization and deteriorated synaptic plasticity (Gao et al. 2009; Rauskolb et al. 2010; Wang et al. 2015). Reduction of BDNF concentration induced in cultured rat hippocampal neurons was related to decreased expression of genes that are functionally related to vesicular trafficking and synaptic communication (Mariga et al. 2015). The pattern of gene expression changes was similar to the profile observed in material coming from patients with Alzheimer’s disease and cognitive impairment.

BDNF has been shown to stimulate cellular proliferation in several brain regions. Its overexpression along with p75NTR binding correlated with generation of neuronal precursors in the olfactory bulb (Young et al. 2007; Bath et al. 2008). BDNF is also involved in regulation of migration of neuronal progenitors along the rostral migratory stream and neuronal settlement in the olfactory bulb (Snapyan et al. 2009). Interestingly, BDNF stimulation led to an increase in neuronal number in the olfactory bulb of rat (Benraiss et al. 2001; Henry et al. 2007). However, the same effect was not observed in mouse (Galvao et al. 2008; Reumers et al. 2008), which may be explained by species-specific differences in regulatory mechanisms.

An interesting effect revealing the practical significance of BDNF is prosurvival enhancement, exerted upon neurons representing dopaminergic, cholinergic, and serotonergic neurotransmitter systems (Foltran and Diaz 2016). Although an explanation for the role of BDNF in this process requires further study, it indicates an important function of this neurotrophin, potentially related to control of neurotransmitter systems and prevention of development of neurodegenerative or psychiatric disorders.

Apart from the influence of BDNF on development of neuronal subpopulations representing different neurotransmitter systems, results of recent studies have demonstrated a stimulatory effect of the serotoninergic system on BDNF/TrkB receptor complex-initiated neurogenesis (Gould 1999). An increase in neuronal proliferation has been reported after administration of serotonin agonists of several receptors, e.g., 5-HT1A (Banasr et al. 2004; Santarelli et al. 2003), 5-HT2B (Diaz et al. 2012), and 5-HT4 (Mendez-David et al. 2014), which may be related to their binding to the BDNF/TrkB receptor complex. This mechanism could explain the proneurogenic effect of antidepressants from the selective serotonin reuptake inhibitor (SSRI) group. These data may stimulate future studies on the mechanisms of action of SSRIs and extend potential indications in therapy for psychiatric and neurodegenerative disorders.

Sotthibundhu et al. reported a stimulatory effect exerted by amyloid β (Aβ) upon neural progenitor cells in the adult subventricular zone (Sotthibundhu et al. 2009). This effect is mediated by Aβ-dependent stimulation of p75NTR, a receptor preferentially binding pro-BDNF, indicating a possible significance of this neurotrophin in neurogenesis. However, this type of Aβ-induced overstimulation of neurogenesis, when occurring in the early stages of development, has been claimed to be responsible for serious disturbances in adult neurogenesis by reducing the number of available neural progenitors.

Another interesting issue that remains to be elucidated is the modulating effect of environmental factors and neuronal activity on the course of BDNF-regulated neurogenesis, which has been documented in numerous publications (Berchtold et al. 2005; Cotman et al. 2007; Vaynman and Gomez-Pinilla 2005). Neurogenesis could be induced by environmental enrichment (Kempermann et al. 1997; Rossi et al. 2006), hippocampus-dependent learning (Gould 1999), and physical exercise (Aimone et al. 2014; Vivar et al. 2013). Although the regulatory mechanisms of neurogenesis induced by these factors are complex and only partially disclosed, increased expression of BDNF was found in each case.

Apart from its role in neurogenesis and neuronal differentiation, BDNF has also been reported to stimulate gliogenesis and glial proliferation during brain development and in some pathological processes (Frisen et al. 1993). Results of animal studies have shown increased expression of the truncated TrkB receptor in the region of reactive gliosis after brain injury. Binding of m-BDNF to the truncated form of TrkB receptor stimulates gliogenesis and differentiation of neural progenitors into glial lineage (e.g., astrocytes) in conditions of reactive gliosis (Cheng et al. 2007). At the same time, however, it enhances the inhibitory effect upon neurogenesis. This interesting aspect of the function of BDNF, in relationship to the neuroglial reactive response, offers promising opportunities related to modulation of the glial response in the course of various neurodegenerative and neurovascular pathologies. However, this potentially effective strategy, based on BDNF-dependent manipulation of the neuroglial response to pathological stimuli, requires further investigation.

## BDNF Modifies Morphological and Functional Aspects of Synaptic Plasticity

The role of BDNF in regulation of synaptic plasticity and learning mechanisms has been extensively studied both in vivo and in vitro (Messaoudi et al. 2002; Minichiello et al. 1999). BDNF influences both functional and structural aspects of synaptic transmission, enhancing activity-induced changes, which leads to increased efficiency of signal transfer (Lynch 2004). The impact of BDNF can be analyzed along several dimensions, with the final results depending on the level of neuronal activity, the isoform of neurotrophin considered (e.g., pro- or m-BDNF), the time period of the action (short- or long-term effects), its localization (pre- versus postsynaptic effects), and cooperation with neurotransmitters, in particular nitric oxide (NO), glutamate (Glu), and GABA, and their receptors.

Silhol et al. reported that learning increases not only BDNF gene expression but also pro-BDNF and TrkB protein level in hippocampus (Silhol et al. 2007). Moreover, increased level of BDNF in mouse hippocampus evoked by voluntary exercises correlated with improved performance in the Morris water maze test and behavioral tasks related to learning (Vaynman et al. 2004). This is in line with reports showing a correlation between physical activity-evoked BDNF overexpression and augmented excitatory transmission (Canossa et al. 1997; Castren et al. 1993; Patterson et al. 1992; Zafra et al. 1990), as well as enhanced synaptic plasticity in the dentate gyrus (Lynch 2004).

According to an interesting hypothesis, the activity-dependent increase of BDNF level is a consequence of stimulation of glutamatergic NMDARs with subsequent Ca^2+^ intracellular influx. This results in activation of CREB and its binding to BDNF promoter, which leads to initiation of transcription (Ghosh et al. 1994; Tao et al. 1998, 2002; Zafra et al. 1991).

Altogether, BDNF expression depends on various forms of cellular and synaptic activity, initiated by stimuli of different modalities. The relationship between BDNF expression level and stimulus-evoked cellular activity reveals reciprocal character. On the one hand, increased expression of BDNF is the result of stimulation, while on the other hand, higher BDNF content strengthens synaptic potentiation, modulates the axo-dendritic morphology, and positively influences neuronal activity.

Numerous studies have shown that m-BDNF expressed after high-frequency hippocampal stimulation enhanced long-term potentiation (LTP) (Chen et al. 1999; Figurov et al. 1996; Kang et al. 1997; Minichiello et al. 1999). In contrast, pro-BDNF expressed during low-frequency stimulation has been reported to induce LTD (Woo et al. 2005; Yang et al. 2014). These observations confirm a close relationship between the chemical structure of the BDNF isoform and the effects of its action in the context of synaptic plasticity. This can also explain the wide range of BDNF-initiated physiological effects exerted by different stimuli.

Molecular processes responsible for hippocampal synaptic potentiation can be categorized, depending on the time course, into short and long term (Abraham 2003; Kandel 2001; Leal et al. 2015; Sweatt 1999), and BDNF can modify both of them. The short-lasting processes controlled by BDNF are based on regulation of neurotransmitter release, modification of preexisting proteins or synapse structure (Leal et al. 2015). The long-term effects are related to changes in gene expression or protein synthesis (Korte et al. 1998; Park and Poo 2013). The BDNF-controlled long-lasting effects of LTP and alterations in the synaptic proteome may result from modulation of microRNA (miRNA) expression (Jaitner et al. 2016; Leal et al. 2014; Smalheiser et al. 2010). Another route for synaptic proteome modification, which is dependent on BDNF-related protein degradation, involves calpains and ubiquitin–proteasome system activation (Leal et al. 2015; Santos et al. 2015).

Apart from this time-based categorization, the influence of BDNF on synaptic plasticity can also be investigated according to the target of its action, i.e., at pre- or postsynaptic elements. Whereas the former is related to regulation of release of neurotransmitters, the latter is concerned with changes in expression of receptors, their molecular characteristics, as well as regulation of signaling pathways (Edelmann et al. 2014).

The presynaptic effect of BDNF on hippocampal LTP has been reported in Schaffer’s collaterals (Zakharenko et al. 2003). This relies on BDNF-induced changes of Glu and GABA release into the synaptic cleft (Figurov et al. 1996). The postsynaptic mechanism of BDNF action has been reported in dentate gyrus (Kovalchuk et al. 2002). In this part of the hippocampus, BDNF modifies the glutamatergic postsynaptic receptors. The m-BDNF/TrkB receptor complex triggers phosphorylation of NR1 and NR2B subunits of NMDA receptor (Caldeira et al. 2007b) and upregulates the GluR1 and GluR2/3 subunits of α-amino-3-hydroxy-5-methyl-4-isoxazolepropionic acid receptors (AMPARs) (Caldeira et al. 2007a; Fortin et al. 2012). These modifications enhance the synaptic strength and initiate LTP in a Ca^2+^ ion concentration dependent manner (Kang et al. 1997; Korte et al. 1998; Messaoudi et al. 2002; Minichiello et al. 1999). It has been revealed that the BDNF-dependent increase in the number of AMPARs in the postsynaptic membrane positively enhances LTP, whereas their elimination results in LTD (Derkach et al. 2007; Fortin et al. 2012). Another mechanism of synaptic strength control relies on BDNF-dependent reduction of GABAA receptor expression and decreased inhibitory GABA-ergic neurotransmission in the hippocampus (Jovanovic et al. 2004).

Apart from the above-mentioned molecular mechanisms involving changes in receptor expression, BDNF also induces some structural modifications, enhancing the activity-related efficiency of synaptic transmission. The BDNF/TrkB receptor signaling cascade triggers processes leading to increase in the number of dendritic spines and increased dendritic arborization, which improves the efficiency of synaptic transmission (Amaral and Pozzo-Miller 2007; Gonzalez et al. 2016; Kumar et al. 2005).

The function of BDNF in modulation of synaptic plasticity is also dependent on its cooperation with neurotransmitters, in particular NO. Although the role of NO in the hippocampal mechanisms of learning and in brain development has been extensively studied, the relationship between NO and neurotrophins involved in these processes remains mostly unknown. Results from recent studies suggest a reciprocal and modulatory relationship between BDNF and NO, which effectively influences synaptic plasticity (Biojone et al. 2015). Binding of BDNF to TrkB receptor upregulates neuronal nitric oxide synthase (nNOS) expression and increases production of NO (Biojone et al. 2015). This effect has been reported in neural progenitors, astrocytes, as well as neocortical and hippocampal neurons (Cheng et al. 2003; Colombo et al. 2012; Kolarow et al. 2014; Sandoval et al. 2011; Xiong et al. 1999).

An interesting hypothesis explaining the role of BDNF and NO in strengthening the synaptic system has been proposed. It has been reported that BDNF-induced increase in NO triggers expression of CREB-dependent genes, which finally results in stimulation of dendritic arborization and enhancement of long-lasting effects of synaptic potentiation (Hardingham et al. 2013; Nott et al. 2008; Riccio et al. 2006). Acting presynaptically, NO can modulate release of Glu and GABA (Steinert et al. 2010). On the contrary, postsynaptic action of NO increases the number of AMPARs, which results in LTP (Malinow and Malenka 2002).

NO-dependent posttranslational modifications of BDNF, such as nitration or *S*-nitrosylation of amino acid residues in the BDNF sequence, negatively change its affinity to TrkB receptor and consequently decrease the impact of BDNF on development of neuronal connections, as well as on synaptic strength as evidenced by LTP (Biojone et al. 2015). Hence, through regulation of NO production, it may be possible to control the BDNF-dependent effects on synaptic plasticity. The physiological significance of BDNF–NO interplay can be attributed to regulation of synaptic strength and elimination of improperly shaped neuronal projections, ultimately resulting in an adequate pattern of connections and maintenance of proper brain functions (Ernst et al. 2000). Better understanding of this relationship requires further research aimed at explaining cognitive, developmental or neuropathological aspects of NO function.

## Conclusions

A large and constantly growing body of evidence indicates involvement of BDNF in numerous neurophysiological processes. In general, the functions of this neurotrophin are related to control of development of neuronal and glial cells, as well as activity-dependent regulation of the synaptic structure and its maintenance, which are critical for memory and cognition. A wide spectrum of processes are controlled by BDNF, exerting sometimes opposite effects in the brain, which can be explained based on the specific pattern of its synthesis, with several biologically active isoforms that interact with different types of receptor, finally initiating a large number of signaling pathways. The physiological role of BDNF, as summarized herein, renders it a potentially valuable tool for many therapeutic strategies. However, clinical applications of this neurotrophin require further intensive study.
